# Attributable causes of chronic kidney disease in adults: a five-year retrospective study in a tertiary-care hospital in the northeast of the Malaysian Peninsula: Causas imputáveis da doença renal crônica em adultos: um estudo retrospectivo de cinco anos de um Hospital de Assistência Terciária no nordeste do Península da Malásia

**DOI:** 10.1590/1516-3180.2015.005

**Published:** 2015-04-14

**Authors:** Muhammad Salman, Amer Hayat Khan, Azreen Syazril Adnan, Syed Azhar Syed Sulaiman, Khalid Hussain, Naureen Shehzadi, Fauziah Jummaat

**Affiliations:** I PharmD, MSc. Doctoral Student, Discipline of Clinical Pharmacy, School of Pharmaceutical Sciences, Universiti Sains Malaysia, Penang, Malaysia, and Lecturer, Department of Pharmacy Practice, University College of Pharmacy, University of the Punjab, Lahore-54000, Pakistan.; II BPharm, MPhil, PhD. Senior Lecturer, Discipline of Clinical Pharmacy, School of Pharmaceutical Sciences, Universiti Sains Malaysia, Penang, Malaysia.; III MD, MMed, FASN. Associate Professor, Chronic Kidney Disease Resource Centre, School of Medical Sciences, Universiti Sains Malaysia, Health Campus, Kota Bharu, Kelantan, Malaysia.; IV BParm, PharmD. Professor, Discipline of Clinical Pharmacy, School of Pharmaceutical Sciences, Universiti Sains Malaysia, Penang, Malaysia.; V BPharm, MPhil, PhD. Professor, University College of Pharmacy, University of the Punjab, Lahore-54000, Pakistan.; VI PharmD, MPhil. Doctoral Student, University College of Pharmacy, University of the Punjab, Lahore-54000, Pakistan.; VII MD, MMed. Lecturer, Department of Gynecology and Obstetrics, School of Medical Sciences, Universiti Sains Malaysia, Health Campus, Kota Bharu, Kelantan, Malaysia.

**Keywords:** Renal insufficiency, chronic, Kidney failure, chronic, Retrospective studies, Hospitals, Malaysia.

## Abstract

**CONTEXT AND OBJECTIVE:**

Chronic kidney disease (CKD) is an escalating medical and socioeconomic problem worldwide. Information concerning the causes of CKD, which is a prerequisite for reducing the disease burden, is sparse in Malaysia. Therefore, this study aimed to evaluate the attributable causes of CKD in an adult population at a tertiary referral hospital.

**DESIGN AND SETTING:**

Retrospective study at Hospital Universiti Sains Malaysia (HUSM).

**METHODS:**

This was an analysis based on medical records of adult patients at HUSM. Data regarding demographics, laboratory investigations, attributable causes and CKD stage were gathered.

**RESULTS:**

A total of 851 eligible cases were included. The patients' mean age was 61.18 ± 13.37 years. CKD stage V was found in 333 cases (39.1%) whereas stages IV, IIIb, IIIa, and II were seen in 240 (28.2%), 186 (21.9%), 74 (8.7%) and 18 (2.1%), respectively. The percentage of CKD stage V patients receiving renal replacement therapy was 15.6%. The foremost attributable causes of CKD were diabetic nephropathy (DN) (44.9%), hypertension (HPT) (24.2%) and obstructive uropathy (9.2%). The difference in the prevalence of CKD due to DN, HPT and glomerulonephritis between patients ≤ 50 and > 50 years old was statistically significant.

**CONCLUSION:**

Our results suggest that DN and HPT are the major attributable causes of CKD among patients at a Malaysian tertiary-care hospital. Furthermore, the results draw attention to the possibility that greater emphasis on primary prevention of diabetes and hypertension will have a great impact on reduction of hospital admissions due to CKD in Malaysia.

## RESUMO

**CONTEXTO E OBJETIVO:**

Doença renal crônica (DRC) é um problema médico e socioeconômico crescente. As informações relativas às causas da DRC são pré-requisito para reduzir a carga da doença, e são escassas na Malásia. Este estudo tem como objetivo avaliar as causas atribuíveis à DRC na população adulta de um hospital de referência terciária.

**TIPO DE ESTUDO E LOCAL:**

Estudo retrospectivo realizado no Hospital Universiti Sains Malaysia (HUSM).

**MÉTODOS:**

Análise de prontuários de pacientes adultos de HUSM. Foram obtidos dados demográficos, exames laboratoriais, causas atribuíveis e estágio da DRC.

**RESULTADOS:**

Um total de 851 casos elegíveis foi incluído. A idade média dos pacientes foi de 61,18 ± 13,37 anos. DRC fase V foi contabilizada em 333 casos (39,1%), enquanto casos de estágio IV, IIIb, IIIa e II foram 240 (28,2%), 186 (21,9%), 74 (8,7%) e 18 (2,1%), respectivamente. A porcentagem de pacientes com DRC estágio V recebendo a terapia de substituição renal foi 15,6%. As causas atribuíveis mais importantes da DRC foram nefropatia diabética (ND) (44,9%), hipertensão (24,2%) e uropatia obstrutiva (9,2%). A diferença na prevalência da DRC devido à ND, hipertensão e glomerulonefrite entre pacientes ≤ 50 anos e > 50 anos de idade foi estatisticamente significativa.

**CONCLUSÃO:**

Nossos resultados sugerem que a ND e a hipertensão são as principais causas atribuíveis da DRC em pacientes sob cuidados terciários na Malásia. Os resultados apontam para a possibilidade de maior ênfase na prevenção primária da diabetes e hipertensão como impactante na redução das internações hospitalares devidas a DRC na Malásia.

## INTRODUCTION

Chronic kidney disease (CKD) is an overwhelming health and socioeconomic problem across the globe. Statistics from the United States suggest that for each patient with end-stage renal disease (ESRD), there are more than 200 with overt CKD (stages III or IV) and nearly 5000 with covert disease (stages I or II).^1^

Deplorably, this sort of evidence is sparse for developing countries, including Malaysia, where the estimated prevalence of CKD is around 9%^2^ and the prevalence of ESRD patients on dialysis has risen from 325 (year 2001) to 975 (year 2012) per million population (pmp). This has been attributed to the escalating incidence of diabetic kidney disease, which accounts for more than half of new ESRD cases.^3^ Information regarding the etiology of CKD stages I to V is scarce in Malaysia. The published data describe only the primary renal disease of ESRD patients.

In one descriptive study by Shaza et al.,^4^ observations were made on 117 ESRD patients receiving treatment at Penang General Hospital, Malaysia, over a period of two and a half years (January 1, 2000, to June 30, 2002). This revealed that the most frequent known causes of ESRD included diabetic nephropathy (DN) and glomerulonephritis (GN), whereas unknown etiologies accounted for 14%. Similarly, Liu and Hooi reported that the major known causes of ESRD incidence in 2003 and 2004 were DN and GN, whereas hypertension (HPT) and obstructive uropathy (OBS) were among the less common causes.^5^ They also reported that there was higher incidence of ESRD due to unknown causes.

## OBJECTIVE

We aimed to describe the demographics, clinical profile and possible attributable causes of CKD among adult patients at a tertiary-care hospital in Malaysia.

## METHODS

A retrospective study based on the medical records of adult patients was conducted at Hospital Universiti Sains Malaysia (HUSM), Kota Bharu, Malaysia, which is a tertiary referral institute located in the state capital of Kelantan. This state has an estimated population of 1.68 million (0.85 million males and 0.84 million females).^6^ HUSM has 33 inpatient units (wards) with a total capacity of 769 beds, and 12 specialist outpatient clinics, including the CKD Resource Centre. The latter serves not only for treatments but also for providing awareness, knowledge and pre-dialysis education to CKD patients from all over the northeast of the Malaysian Peninsula (states of Kelantan, Terengganu and Pahang). 

Permission to conduct this study was acquired from the Human Research Ethics Review Committee, Universiti Sains Malaysia. Medical records (paper-based records) of adult patients (age ≥ 18 years) either seen in the CKD Resource Centre or admitted to HUSM due to CKD, between January 2009 and December 2013, were eligible for inclusion in the study. Medical records relating to patients with acute kidney injury, patients with acute deterioration on chronic kidney disease with insufficient information to confirm the attributable cause and/or stage of CKD and patients aged < 18 years were excluded. 

The researcher extracted the data from the medical records between January and May 2014. An average of 20 medical files were reviewed per day and information regarding demographics, laboratory findings, patient's medical history, family history of diseases, ultrasound or X-ray results relating to kidney, ureter and bladder (KUB), chest radiograph results, types of renal replacement therapy and probable cause of the disease were gathered.

### Criteria for assessment of the attributable causes of chronic kidney disease

The attributable causes of CKD were assessed using the diagnosis that had been made by the nephrologist or clinician. If this was unavailable, the criteria described below were used to determine the probable cause of CKD.

DN was considered to be the underlying cause of CKD if a patient had a confirmed diagnosis of diabetes mellitus (DM) and any of the following criteria: long-standing DM before the onset of CKD (minimum 10 years), evidence of substantial proteinuria, presence of diabetic retinopathy and normal-sized kidneys on ultrasound.^7^

HPT was diagnosed as a cause of CKD if a patient had any of the following conditions: long history of hypertension predating renal impairment (minimum of five years), presence of left ventricular hypertrophy or hypertensive retinopathy and no substantiation of any other etiology of kidney disease.^8^

Patients with chronic renal failure who had pathological proteinuria and normal or small kidneys and who ultimately developed end-stage renal disease (ESRD) with small kidneys were categorized as cases of chronic glomerulonephritis, whether or not a biopsy had been obtained.^9^

The procedures used for diagnosing obstructive uropathy (OBS) were KUB ultrasound, KUB radiography, cystoscopy, intravenous pyelography, retrograde pyelography and radioisotope renography. This diagnosis was made if the patient had solid evidence of urinary obstruction (nephrolithiasis, ureterolithiasis, cystolithiasis, benign prostatic hyperplasia, urethral stricture, etc.), with or without hematuria and presence of hydronephrosis and hydroureter.

Chronic pyelonephritis (CPN) was considered to be the possible underlying etiology of CKD if a patient had a history of recurrent or persistent urinary tract infections and noticeable renal scarring.^9^

The diagnosis of adult polycystic kidney disease (APKD) was made if a patient had enlarged or palpable kidneys with bilateral complex renal cysts, with or without a family history of polycystic kidney disease and a history of flank pain.

Toxic nephropathy was considered to be the presumptive cause of CKD if a patient had a strong history of use of nephrotoxic agents (e.g. nonsteroidal anti-inflammatory drugs (NSAIDs), traditional medicines etc.) and in the absence of indicators of any other etiology of kidney disease.

Patients who were diagnosed with or presented CKD for the first time, for which no apparent cause could be found, were categorized as having disease of unknown cause. Other attributable causes were diagnosed on the basis of kidney imaging, renal Doppler ultrasonography, renal angiography and renal biopsy results.

The CKD was classified based on the estimated glomerular filtration rate (GFR) as per the Kidney Disease Improving Global Outcomes (KDIGO) guidelines.^10^ The GFR was calculated by using the Chronic Kidney Disease Epidemiology Collaboration (CKD-EPI) equation.^11^

The data are presented as mean ± standard deviation (SD) for continuous variables and numbers and/or percentages for categorical variables. The data were analyzed according to age groups (≤ 50 or > 50 years of age) and gender. Comparisons of categorical and continuous variables were assessed by means of the chi-square test and t test, respectively. Variables that were not available for a large proportion of the sample were not included in the final analysis. The analyses were carried out by using the Statistical Package for the Social Sciences (SPSS version 20, Chicago, IL, USA) for Windows. A Pvalue < 0.05 was considered statistically significant.

## RESULTS

The total numbers of patients admitted to and visiting HUSM during 2009-2013 are shown in Table 1. Eight hundred and fifty-one eligible cases (541 males and 310 females) were included in the present study. The sociodemographic data are shown in Table 2. The patients' mean age was 61.18 ± 13.37 years (range 18-92), with preponderance of patients (32.4%) in the age group of 60-69 years and only 3.2% in the age group of 18-29 years. There was predominance of Malay race (95.9%), while Chinese, Indian and other races constituted the minority. Approximately 91% of our cases were from the state of Kelantan and 69.2% were from urban areas. It was found that 7.4% of the patients were current smokers, while 30.7% were former smokers. Twenty-three patients (2.7%) had a family history of CKD, whereas family histories of DM, hypertension and heart disease were noticed in 13.3%, 10.6% and 7.9% of the patients, respectively. There were 98 cases with histories of urolithiasis. Among all the patients, 21.5% were chronic users of nonsteroidal anti-inflammatory drugs (NSAIDs) and 3.5% self-reported use of traditional medicine. The mean estimated GFR was 23.16 ± 16.71 ml/min/1.73m^2^, as calculated using the CKD-EPI equation. Using the KDIGO classification for CKD staging, we found that our patients were predominantly in the late stages of CKD (stages IV and V).

**Table 1:**
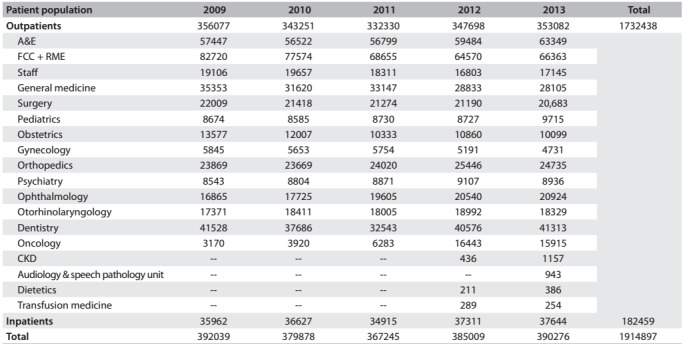
Patient population at Hospital Universiti Sains Malaysia between 2009 and 2013

A&E = Accident & Emergency Department; FCC = Family Care Clinic; RME = Rapid Medical Evaluation; CKD = Chronic Kidney Disease Resource Centre.

**Table 2:**
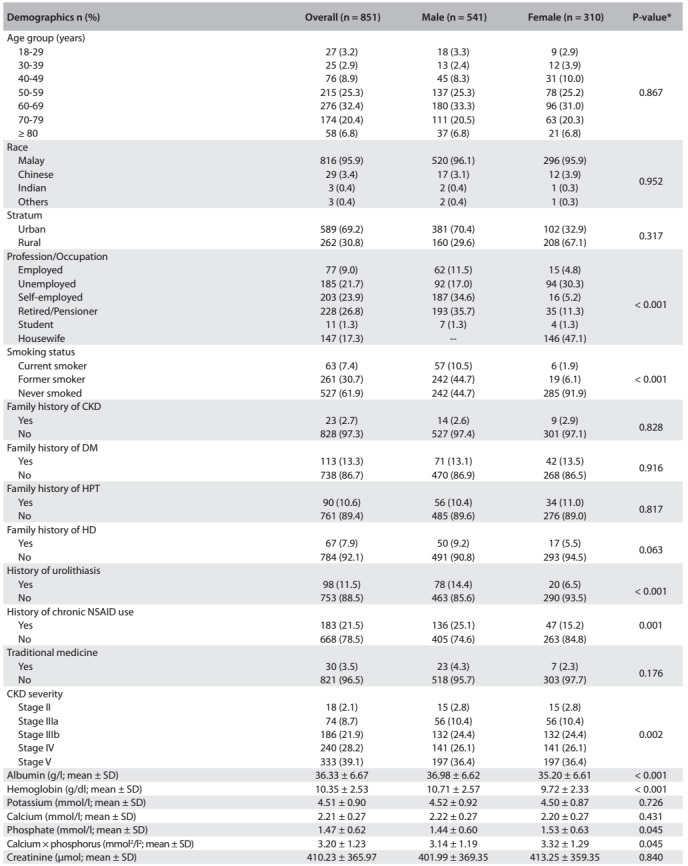
Patients' demographic data obtained from the sample

CKD = chronic kidney disease; DM = diabetes mellitus; HPT = hypertension; HD = heart disease; NSAID = nonsteroidal anti-inflmmatory drug

The majority of the patients were asymptomatic. However, the chief complaints at presentation were nausea (20.9%), vomiting (15.3%), lethargy (52.6%), malaise (52.9%), edema (36.1%), poor appetite (50.3%), weight loss (11.6%), dyspnea (34.7%), decreased urine output/oliguria (10.8%) and dysuria (5.1%). 

The most frequent attributable cause of CKD was DN (44.9%), followed by HPT (24.2%) and OBS (9.2%), as shown in Table 3. The cause of CKD was unknown in 9.4% of our cases. Out of 57 patients with glomerular disease, five had focal segmental glomerulosclerosis (FSGS), two had IgA nephropathy, five had lupus nephritis (LN) and the rest had chronic glomerulonephritis. Of the seven in the miscellaneous group, two patients had CKD due to renal tubular acidosis (one patient with distal tubular acidosis and another one with proximal tubular acidosis), one had renal artery stenosis, three had congenital urogenital malformations and one had CKD due to trauma/injury to the kidneys.

**Table 3:**
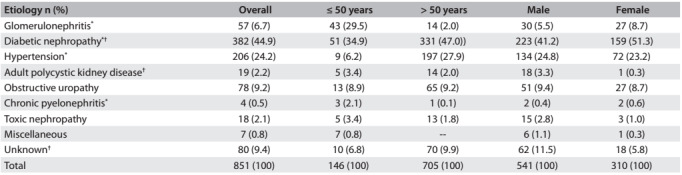
Attributable causes of chronic kidney disease in the sample

*Signifiant diffrence (P < 0.05) between age categories; †signifiant diffrence (P < 0.05) between male and female

There was a substantial statistical difference in the prevalence of CKD due to DN between younger patients and older patients (34.9% versus 47.0%; P = 0.008). Similarly, the difference in prevalence of CKD due to GN and HPT between the younger and older age groups was statistically significant (29.5% versus 2.0%; P < 0.001; and 6.2% versus 27.9%; P < 0.001, respectively). The differences in the prevalence of CKD between male and female patients, due to DN (41.2% versus 51.3%; P = 0.004), APKD (3.3% versus 0.3%; P = 0.004) and unknown cause (11.5% versus 5.8%; P = 0.007), were also statistically significant.

**Figure 1:**
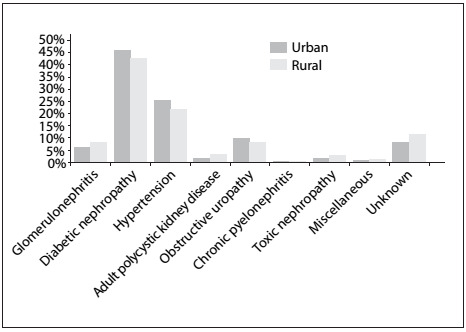
Attributable causes of chronic kidney disease in rural and urban areas.

The attributable causes of CKD in the urban and rural zones are depicted in Figure 1. These results showed that the place where the patients lived (rural or urban areas) had no statistical influence on the prevalence of CKD due to any of the etiologies. Increased renal parenchymal echogenicity was the leading ultrasound abnormality (43.5%), while atrophic kidneys were seen in 17.6% of the patients. Increased cardiothoracic ratio (cardiomegaly) was found in 52.6% of the subjects. Only 8.1% and 3.9% of the patients had pleural effusion and pulmonary edema, respectively.

Renal replacement therapy, consisting of either hemodialysis (HD) or peritoneal dialysis (PD), was used in 134 patients (15.6%). Among these patients, HD was the most frequent type of treatment (93.3%), while continuous ambulatory PD was used much less frequently (6.7%). None of the patients received continuous cycling peritoneal dialysis or kidney transplants. In our centre, the majority of the patients decide themselves which type of renal replacement therapy they want. To help these patients to decide, pre-dialysis talks are held at the Chronic Kidney Disease Resource Centre, Hospital Universiti Sains Malaysia. After patients acquire all the information about types of renal replacement therapy, they choose their treatment. Patients who are interested in kidney transplantation are referred to Hospital Kuala Lumpur.

## DISCUSSION

Our main results showed that most of the patients with CKD in the sample were men and older people with advanced chronic kidney disease (stages IV and V). Beyond that, the most frequent attributable causes of CKD in the sample were DN and HPT. 

The mean age of the patients in the present study was similar to that of previous reports.^12^ ^13^ Higher prevalence of CKD in the elderly population has been attributed to the fact that renal function declines with age.^14^ The ethnic composition of Malaysia is Bumiputera (Malay) (67.4%), Chinese (24.6%), Indian (7.3%) and others (0.7%).^15^ However, the predominance of Malay patients (95.9%) in our study population was because the majority of our patients were from the states of Kelantan and Terengganu in Malaysia, where the dominant ethnic group is Malay. The higher percentage of former smokers and lower percentage of current smokers in the present study was because the CKD patients were advised to quit smoking, given that it causes rapid progression of kidney disease.^16^ The level of chronic NSAID use (21.5%) in the current study was slightly higher and the level of traditional medicine use (3.5%) was lower than that reported previously.^2^ This history of higher NSAID use might be associated with gout (16.3%). A gender-based comparison of demographics revealed that there was no significant difference in age groups, race and habitation between male and female CKD patients. These findings demonstrated that the abovementioned factors had no impact on the prevalence of CKD among male and female patients. The majority of the patients were in the late stages of CKD at the time of presentation to the study setting. Similar to these findings, Al-Ramahi also found higher prevalence of CKD stage V subjects in their study conducted at Penang General Hospital, Malaysia.^13^ The higher prevalence of the late stages of CKD might be attributable either to late nephrology referral by primary care physicians or lack of awareness among patients themselves, regarding the proper time to seek medical attention. 

Regarding the preferred types of dialysis, PD is the favorite treatment method in Mexico^17^ and Hong Kong,^18^ but HD is the major mode of treatment in various countries including Malaysia.^19^ ^20^ ^21^ Consistent with these findings, we also observed that the majority of ESRD patients preferred HD over PD. In Malaysia, PD is underutilized, notwithstanding the efforts of the Malaysian government to promote this treatment method. This can mainly attributed to the abundance of HD centers provided by the private sector and non-governmental organizations (NGOs), as well as the better survival rates among HD patients.^21^ None of our patients received continuous cycling peritoneal dialysis or kidney transplants, which might have been due to financial constraints. 

DN was the foremost attributable cause of CKD in our study population, and this finding is similar to previous reports regarding the causes of CKD (stages III to V).^7^ ^8^ The second leading cause of CKD was HPT (24.2%), and the occurrence rate of HPT was higher than that reported in Sri Lanka. 7 8 This finding demonstrates that there is a need to improve the management of hypertension, since this is a major contributor not only to renal disease and its progression towards ESRD but also to cardiovascular morbidity and mortality.^22^ The percentage of GN in our study was less than the previously reported 9.9% to 12%.^7^ ^8^ This may have been because infectious diseases are more prevalent in countries of low economic level, given their poor sanitation, insufficient supply of safe water and greater concentrations of disease-transmitting vectors.^23^ The prevalence of kidney disease due to nephrotoxic drugs was 2.1%, which reflects the need for awareness and proper knowledge in relation to excessive use of these agents. The cause of CKD was unknown in 9.4% of the patients, which was comparable to the findings of a recent report.^8^ However, this rate was considerably less than that reported by Gooneratne et al.^7^ and Athuraliya et al.^24^ The decline in the prevalence of unknown causes may have been due to better diagnostic work-up, thus resulting in a greater number of patients diagnosed with an exact cause for their disease, such as glomerular diseases, heredofamilial causes and toxic nephropathy. 

DN and HPT were the leading contributors towards CKD in the older age category (47.0% and 27.9%, respectively). The differences in the frequencies of CKD due to DN and HPT between the younger and older age groups were statistically significant, and this finding was comparable to the results from another study conducted in an Asian country.^8^ Gender-related differences in health habits or lifestyle may cause differences in the risk factors for developing DM and, consequently, may cause variations in DM prevalence between men and women.^25^ Previous studies have also shown that the prevalence of DM is higher in the female than in the male population.^26^ ^27^ This might be the reason why the prevalence of CKD due to DN was significantly higher among females than among males in the current study. The frequency of GN was considerably higher in the younger than in the older age category (P < 0.001), which was similar to the findings of Wijewickrama et al.^8^

Although we achieved the desired objectives of our study, it had some limitations. Firstly, this study was conducted in a single tertiary referral hospital in the northeast of the Malaysian peninsula and only 2.2% of the patients were from outside the northeastern region. Therefore, our results may not give a clear picture of attributable causes of CKD throughout Malaysia. Secondly, due to the predominance of the Malay ethnic group, the findings of this study cannot be generalized to the overall Malaysian population. Thirdly, due to the retrospective nature of this study, some data were not fully available: for example, income status, health-seeking behavioral patterns, distance to the healthcare facility and ability to afford transportation. However, this may not have significantly influenced the results. Lastly, we did not use a probability sampling method, e.g. random sampling, and therefore we had disadvantages such as selection bias and non-generalizability.

This study recommends that emphasis is required at two levels: firstly, identification of individuals at risk of CKD development or presenting early CKD, as soon as possible; and secondly, early referral of CKD patients to nephrology centers by primary care physicians. This second approach has already demonstrated improvements in treatment outcomes, and it results in earlier preparation of the start of dialysis.^28^ Moreover, education programs and seminars need to be conducted in order to increase awareness among CKD patients regarding dietary habits, nephrotoxic agents, smoking cessation and choice of types of renal replacement therapy etc.

## CONCLUSION

The data suggest that DN and HPT are the major attributable causes of Chronic Kidney Disease among patients at a tertiary referral hospital in the northeast of the Malaysian peninsula. Although the results cannot be easily generalized to the general population, the study draws attention to the possibility that greater emphasis on primary prevention of diabetes and hypertension will have a large impact on reduction of hospital admissions due to CKD in Malaysia.
